# Reprogramming Extracellular Vesicles for Protein Therapeutics Delivery

**DOI:** 10.3390/pharmaceutics13060768

**Published:** 2021-05-21

**Authors:** Leyla A. Ovchinnikova, Stanislav S. Terekhov, Rustam H. Ziganshin, Dmitriy V. Bagrov, Ioanna N. Filimonova, Arthur O. Zalevsky, Yakov A. Lomakin

**Affiliations:** 1 Shemyakin-Ovchinnikov Institute of Bioorganic Chemistry RAS, 117997 Moscow, Russia; leyla_ovchinnikova@yahoo.com (L.A.O.); sterekhoff@mail.ru (S.S.T.); ziganshin@mail.ru (R.H.Z.); ioannishka@gmail.com (I.N.F.); aozalevsky@gmail.com (A.O.Z.); 2 Department of Chemistry, Lomonosov Moscow State University, 119991 Moscow, Russia; 3 Faculty of Biology, Lomonosov Moscow State University, 119234 Moscow, Russia; bagrov@mail.bio.msu.ru; 4 Phystech School of Biological and Medical Physics, Moscow Institute of Physics, Technology (National Research University), 141701 Dolgoprudny, Russia

**Keywords:** extracellular vesicles, exosomes, EVs, protein delivery, nanocages, VSV-G, enveloped viruses, macromolecule delivery, mass spectrometry

## Abstract

Delivering protein therapeutics specifically into target cells and tissues is a promising avenue in medicine. Advancing this process will significantly enhance the efficiency of the designed drugs. In this regard, natural membrane-based systems are of particular interest. Extracellular vesicles (EVs), being the bilayer lipid particles secreted by almost all types of cells, have several principal advantages: biocompatibility, carrier stability, and blood–brain barrier penetrability, which make them a perspective tool for protein therapeutic delivery. Here, we evaluate the engineered genetically encoded EVs produced by a human cell line, which allow efficient cargo loading. In the devised system, the protein of interest is captured by self-assembling structures, i.e., “enveloped protein nanocages” (EPN). In their turn, EPNs are encapsulated in fusogenic EVs by the overexpression of vesicular stomatitis virus G protein (VSV-G). The proteomic profiles of different engineered EVs were determined for a comprehensive evaluation of their therapeutic potential. EVs loading mediated by bio-safe Fos–Jun heterodimerization demonstrates an increased efficacy of active cargo loading and delivery into target cells. Our results emphasize the outstanding technological and biomedical potential of the engineered EV systems, including their application in adoptive cell transfer and targeted cell reprogramming.

## 1. Introduction

Delivering protein cargoes into target cells remains a significant challenge for modern pharmaceuticals and cell biology still holding a promise of a new era of precision medicine. Targeted delivery technology aims at creating multifunctional carriers that are capable of circulating in the patient’s body for a long time and concurrently have low toxicity [1]. In light of this, extracellular vesicles (EVs) seem to be the perfect carriers for protein therapeutics. EVs are classified into two main subtypes: ectosomes (or microvesicles) and exosomes [2]. These two types of vesicles differ in size, origin, composition, and biogenesis mechanism [3]. Generally, exosomes are characterized as nanosized (40–160 nm) EVs, originating from multivesicular bodies and secreted by cells [4]. Exosomes can be isolated by ultracentrifugation at 100,000× *g.* Ectosomes (microvesicles) are much bigger and have a broader size distribution (100–1000 nm) and plasma membrane origin. They are released during cell-surface budding [5] and can be precipitated at 10,000× *g* [6]. Apoptotic bodies (>1000 nm) are often classified as the third type of EVs. They are highly specific and make up a minor group of EVs formed during the late stages of apoptosis [7].

Naturally occurring EVs are ubiquitous in all body fluids [8] and are secreted by almost all types of cells [9]. They perform important functions of signal transmitting between cells, exchanging genetic information and eliminating dangerous substances from the cell [10]. EVs are capable of encapsulating various macromolecular cargoes such as proteins, nucleic acids and lipids [11,12]; in addition, various receptors and markers may be present on their surface. Although early works described them as cells’ by-products [13], a lot of studies report the significant role of EVs as biomarkers for a variety of human pathological conditions nowadays [14,15,16,17,18]. The major advantages of EVs as therapeutic carriers encompass: (i) natural biocompatibility as their envelope is formed from the same cell membrane [19,20], (ii) the ability to pass through the blood–brain barrier [21,22] and (iii) their versatility as a therapeutic tool since surface receptors could be easily modified [23,24]. Furthermore, harnessing the autologous EVs allows the evasion of the immunogenic response [25]. Taken together, all these benefits make EVs a promising drug carrier, but applying them in therapeutic practice is still restricted. The main limiting factors for EVs application include the difficulties associated with the standardization of the resulting vesicles, variations in the non-specifically encapsulated cellular proteins, and a yet insufficiently effective loading technique [26]. The quantity of naturally occurring EVs is often small and the existing methods for the isolation of EVs still provide low yields. This calls for designing EVs that could overcome these limitations.

Herein, we investigate the engineered EVs, capable of delivering protein cargoes in vitro and in vivo. We improved the engineered EVs to achieve higher delivery efficacy and biocompatibility, adapting this system further for human treatment. The core element of the previously published engineered EVs is “enveloped protein nanocages” (EPN), performing three different functions essential for the release from eukaryotic cells within membrane envelopes: membrane binding, self-assembly, and the recruitment of the endosomal sorting complexes required for transport (ESCRT) machinery [27,28]. This protein can self-assemble into highly ordered 60-subunit dodecahedron scaffolds and release from the cell enveloped in cellular membrane upon additional expression of vesicular stomatitis virus G glycoprotein (VSV-G). Each protein EPN nanocage can be actively loaded with up to 60 molecules of therapeutic protein, increasing EV capacity. Despite the obvious benefits described EVs have, this system contains the toxic fragment of viral protein R (Vpr) [29,30], required for active protein loading. It thus raises serious concerns about their safety once applied in therapeutics.

Here, we develop a novel technique of EPN loading, based on the dimerization of Jun and Fos domains. In our system, the cargo protein is fused with the Jun domain while each EPN monomer contains the Fos domain (Figure S1). The Jun–Fos dimerization provides active loading of EVs with cargo proteins. An increased protein loading is achieved via the flexibility of the Fos domain linker and the overall compactness of the resultant structure. The Fos–Jun heterodimer, in comparison with the Vpr protein, takes up less space inside the EVs and provides more efficient cargo loading. In order to achieve a more uniform distribution of genetic material and, consequently, the release of the fully loaded EVs, we created a lentiviral construct and highlighted the advantages of using it. To better understand their molecular composition, we have characterized the morphology and proteome of engineered EVs. Furthermore, we demonstrate a more effective protein delivery into the target cells harnessing the modified engineered EVs.

## 2. Materials and Methods

### 2.1. Cell Lines

HEK293T and Jurkat cells were cultured, respectively, in DMEM or RPMI media supplemented with 10% FBS (*v*/*v*) and L-Glutamine (all Gibco, OK, USA). HEK293F cells were cultured in FreeStyle medium (Gibco, OK, USA) while shaking at 125 rpm. All cell lines were maintained in a humidified incubator at 37 °C with 5% CO_2_. Cells were tested for mycoplasma contamination using MycoReport kit (Evrogen, Moscow, Russia).

### 2.2. Plasmids

All original plasmid constructs are accessible through the Addgene plasmid repository (https://www.addgene.org, accessed on 30 March 2021). Initial EPN containing vector [28] was obtained from Addgene (Addgene ID: 80046). The pET-EPN-His-p6, pET-EPN-Fos-C, pET-EPN-Fos-M, pET22-NanoLuc (Addgene ID: 167310) and pET22-NanoLuc-Jun (Addgene ID: 167312) prokaryotic expression constructs were generated by PCR amplification of the coding sequences and transferred into a pET22-based vector, using *NdeI* and *BlpI* restriction sites. Glycine linker was added for conformational delimitation and His(6)-tag for detection and affinity purification. The pCMV-EPN-Fos-C (Addgene ID: 167306), pCMV-EPN-Fos-M (Addgene ID: 167307) and pCMV-NanoLuc-Jun (Addgene ID: 167308) mammalian expression constructs are based on pCMV vector and were obtained using *NotI*/*XhoI or HindIII*/*KpnI* restriction sites for the cloning. The pDONR-NanoLuc-EPN-VSVG (Addgene ID: 167305) vector is based on a donor vector pDONR. In this construct, the genes coding NanoLuc fused with Jun domain, EPN fused with Fos domain and VSV-G tagged with 3xFLAG are separated with two IRES (Internal Ribosome Entry Site) sequences. The final lentiviral vector pLX-NanoLuc-EPN-VSVG (Addgene ID: 167304) was obtained on the basis of pDONR-NanoLuc-EPN-VSVG vector using Gateway technology (Life Technologies, Carlsbad, CA, USA). PCR was performed on a Thermal cycler T100 (BioRad, Berkeley, CA, USA) using Q5 polymerase (NEB, Hitchin, UK).

### 2.3. Protein Expression in E. coli and Purification

Protein expression of prokaryotic EPN (pET-EPN-His-p6, pET-EPN-Fos-C, pET-EPN-Fos-M) and NanoLuc (pET22-NanoLuc, pET22-NanoLuc-Jun) was performed in *E. coli* cell strain BL21 DE3 as previously published [31] with several minor changes. Briefly, the overnight culture was inoculated into 2xYT medium at 1:100 (*v*/*v*) with ampicillin (100 μg/mL) and 2% glucose added. Cell culture was grown at 37 °C to an OD_600_ of 0.4–0.5. After the medium changed to glucose-free, an inducer (isopropyl-β-D-1-thiogalactopyranoside, IPTG) was added at a concentration of 0.5 mM. The expression culture was grown for 4 h at 30 °C. Collected pellets were resuspended in 25 mM Tris-HCl (pH 8.0), 500 mM NaCl buffer (for EPN proteins) or 50 mM NaH_2_PO_4_, 300 mM NaCl, 10 mM Imidazole buffer (for NanoLuc proteins) with an addition of cOMPLETE protease inhibitor cocktail (Roche, Mannheim, Germany) and 0.2 mg/mL of lysozyme. Cells were disrupted by sonication, clarified lysates were centrifuged at 20,000× *g* for 20 min and filtrated through a 0.45 μm membrane (Durapore, Darmstadt, Germany).

The His-tagged protein was purified using a Ni-NTA resin (Qiagen, Hilden, Germany) column with 25 mM Tris-HCl (pH 8.0), 500 mM NaCl, 30 mM imidazole as running/wash buffer and 25 mM Tris-HCl (pH 8.0), 500 mM NaCl, 500 mM imidazole as elution buffer (for EPN protein). After dialysis against 25 mM Tris-HCl (pH 8.0), 500 mM NaCl buffer and concentration steps, size-exclusion chromatography was performed at a rate of 0.5 mL/min on Superose 6 10/300 (GE Healthcare) columns using 25 mM Tris-HCl (pH 8.0), 500 mM NaCl or 3 M GITC, 25 mM Tris-HCl (pH 8.0), 500 mM NaCl as running buffer.

Alternatively, His-tagged NanoLuc-Jun protein was purified using a Ni-NTA resin in 50 mM NaH_2_PO_4_, 300 mM NaCl, 10 mM Imidazole as running buffer, 50 mM NaH_2_PO_4_, 300 mM NaCl, 20 mM Imidazole as wash buffer and 50 mM NaH_2_PO_4_, 300 mM NaCl, 250 mM Imidazole as elution buffer. After overnight dialysis against 50 mM NaH_2_PO_4_, 300 mM NaCl buffer, protein concentration was measured and luciferase activity was determined through NanoGlo Luciferase Assay System (Promega, Madison, WI, USA) under manufacturer’s protocol.

### 2.4. Dynamic Light Scattering

All dynamic light scattering (DLS) measurements were obtained using Zetasizer Nano ZS (Malvern Instruments Ltd., WR, Malvern, UK). Purified protein nanocages were measured at 25 °C, then the temperature was ramped up to 60 °C, then ramped back down to 25 °C. Measurements were taken in the presence of TBS: 25 mM Tris, 500 mM NaCl or buffered GITC: 25 mM Tris, 500 mM NaCl, 3 M GITC. Each sample was allowed to equilibrate in the respective buffer for at least 24 h before measurement.

### 2.5. Isolation and Purification of EVs

To optimize EV production, HEK293T and HEK293F cell lines were used. HEK293F cells were seeded in a 24-well plate at 0.5 × 10^6^ cells/mL to a final volume 1 mL per well. HEK293T cells were seeded in a 12-well plate. After 24 h, HEK293F reached a final density of 1 × 10^6^ cells/mL, and HEK293T reached 80–90% confluence and were transfected with: (i) 0.5 μg of pCMV-NanoLuc-Jun, 0.9 μg of pCMV-EPN-Fos-C, 0.1 μg of pCMV-VSVG or (ii) 0.9 μg of pCMV-EPN-Fos-C, 0.1 μg of pCMV-VSVG or (iii) 0.5 μg of pCMV-NanoLuc-Jun using 293fectin transfection reagent (Gibco, OK, USA) for HEK293F or Lipofectamine 3000 transfection reagent (Invitrogen, MA, USA) for HEK293T. The plasmid ratio was defined in the previously published protocol for the production of engineered EVs [28]. The culture supernatant was collected 48 h and 72 h after transfection. Next, the cell debris and microvesicles were removed after two steps of differential centrifugation at 4 °C (300× *g* for 10 min, 1000× *g* for 20 min) and subsequent filtration of supernatant through 0.45 μm filter device (GE Healthcare, UK). EVs were concentrated using Amicon Ultra-0.5 mL 100 kDa Filter Unit (Millipore, Cork, Ireland) and washed with PBS (Gibco, OK, USA) three times. To determine the quantity of NanoLuc luciferase in EVs, Nano-Glo Luciferase Assay (Promega, Madison, WI, USA) was performed under manufacturer’s protocol.

For large-scale production of the genetically encoded EVs, HEK293T cells were seeded in T-75 flasks. After 24 h, cells reached 80–90% confluence and were transfected with appropriate plasmids (Table S1) using Lipofectamine 3000 transfection reagent. After 6 h, cells were washed with PBS to remove vesicle residues from FBS and the media were changed to DMEM supplemented with 0.5% BSA (Sigma-Aldrich, St. Louis, MO, USA) and L-Glutamine. Forty-eight hours after transfection, EV-containing media were collected and cell debris and microvesicles were depleted as described above. Purified EV-containing concentrate was collected through sucrose density gradient ultracentrifugation. Briefly, EVs were mixed with 90% (*w*/*v*) sucrose to a final sucrose percentage of 82% (*w*/*v*) and then overlaid with a sucrose gradient (10% (*w*/*v*)—70% (*w*/*v*) sucrose in PBS). Centrifugation was performed at 100,000× *g* (RCF avg) for 16 h at 4 °C in Beckman Coulter L8-M ultracentrifuge, using Beckman Coulter SW 28 Ti Swinging-Bucket rotor. Fractions, enriched with EVs, were determined using NanoGlo Luciferase Assay, mixed and diluted with PBS, then concentrated using Amicon Ultra-15 centrifugal Filter unit with 100 kDa cut-off (Millipore, Cork, Ireland) and washed with PBS three times. Protein concentrations in probes were measured by CBQCA Protein Quantitation Kit (Invitrogen, Carlsbad, CA, USA). The diameters of EVs were determined in PBS at 25 °C using a Zetasizer Nano ZS.

### 2.6. Western Blot Analysis

A total of 1 μg of EVs (total protein) was lysed in RIPA buffer (50 mM Tris-HCl (pH = 8), 150 mM NaCl, 1% (*w*/*v*) Nonidet P-40, 0.25% (*w*/*v*) Na-deoxycholate, 0.1% (*w*/*v*) SDS, 1 mM PMSF) and incubated at RT for 5 min. Then, EV samples were mixed with Laemmli buffer (125 mM Tris-HCl (pH = 6.8), 4% (*w*/*v*) SDS, 10% (*w*/*v*) 2-mercaptoethanol, 20% (*w*/*v*) glycerol, 0.004% (*w*/*v*) bromphenol blue) in ratio 1:1 (*v*/*v*) and heated for 5 min at 95 °C. Proteins were resolved on 12% PAGE and transferred to a 0.45 μm nitrocellulose membrane (Amersham, Darmstadt, Germany). The membrane was then blocked in 5% (*w*/*v*) non-fat dry milk in PBS with 0.05% (*v*/*v*) Tween-20 (Helicon, Moscow, Russia). For chemiluminescence detection of proteins, monoclonal HRP-conjugated anti-FLAG M2 antibodies (Sigma-Aldrich, St. Louis, MO, USA) in a 1:20,000 dilution and Clarity Western ECL Blotting Substrate (Bio-Rad Laboratories Inc., Hercules, CA, USA) were used.

### 2.7. Transmission Electron Microscopy (TEM)

The carbon-coated TEM grids (Ted Pella, Redding, CA, USA) were treated using a glow discharge device Emitech K100X (Quorum Technologies Ltd., Lewes, UK) at 25 mA current for 45 s. This treatment turned the carbon surface hydrophilic and increased the EVs’ adsorption. The suspension of EVs was deposited onto the grid for 2–3 min, and the liquid was blotted using filter paper (OdiChem, Moscow, Russia). The grids were quickly transferred onto the drops of 1% uranyl acetate, stained for 1–2 min, blotted and dried. Images were obtained using a JEM-1011 (Jeol, Tokyo,Japan) transmission electron microscope equipped with Orius SC1000W camera (Gatan Inc., Pleasanton, CA, USA). The magnification was in the range from 30,000× to 250,000×; the acceleration voltage was 80 kV. The sizes of the EVs were measured using ScanEV [32].

### 2.8. Protein Delivery of EV Cargoes to Target Cells

The ability of engineered EVs to deliver NanoLuc-Jun into target cells was determined using NanoGlo Luciferase Assay System (Promega, Madison, WI, USA). EVs were purified by sucrose density centrifugation as described above. EVs were aligned according to the portion of NanoLuc protein in each sample (375 pg NanoLuc), added to 300,000 Jurkat cells and incubated for 2 h in serum-free medium in triplicate. Recombinant NanoLuc-Jun protein was added to control wells. After incubation, the cells were washed with PBS at 300× *g* for 10 min and then were incubated with 0.005% NP-40 with rotation at room temperature for 10 min. After incubation with detergent, cells were washed twice with PBS and 40,000 cells from each sample were analyzed by Nano-Glo Luciferase Assay (Promega, Madison, WI, USA) to determine the quantity of delivered protein in target cells.

### 2.9. LC-MS/MS and Bioinformatics

Samples were loaded to a home-made trap column 100 × 0.1 mm, packed with Inertsil ODS3 3 μm sorbent (GL Sciences), in the loading buffer (2% (*v*/*v*) acetonitrile, 0.1% (*v*/*v*) trifluoroacetic acid) at 4 µL/min flow and separated at RT in a home-packed [33] fused-silica column 300 × 0.1 mm packed with Reprosil PUR C18AQ 1.9 (Dr. Maisch) into an emitter prepared with P2000 Laser Puller (Sutter, Atlanta, GA, USA). Reverse-phase chromatography was performed with an Ultimate 3000 Nano LC System (Thermo Fisher Scientific, Waltham, Ma, USA), which was coupled to the Q Exactive Plus Orbitrap mass spectrometer (Thermo Fisher Scientific) via a nanoelectrospray source (Thermo Fisher Scientific). Peptides were loaded in a loading solution (98% 0.1% (*v*/*v*) formic acid, 2% (*v*/*v*) acetonitrile) and eluted with a linear gradient: 3–35% solution B (0.1% (*v*/*v*) formic acid, 80% (*v*/*v*) acetonitrile) for 80 min; 35-55% B for 10 min, 55% B for 2 min, 55–99% B during 0.1 min, 99% B during 2 min, 99–2% B for 0.1 min at a flow rate of 500 nL/min. After each gradient, the column was reequilibrated with solution A (0.1% (*v*/*v*) formic acid, 3% (*v*/*v*) acetonitrile) for 10 min. MS1 parameters were as follows: 70K resolution, 320–1600 scan range, max injection time—30 ms, AGC target—3 × 10^6^. Ions were isolated with 1.4 m/z window, preferred peptide match and isotope exclusion. Dynamic exclusion was set to 30 s. MS2 fragmentation was carried out in HCD mode at 17.5K resolution with HCD collision energy 29%, max injection time—50 ms, AGC target—1 × 10^5^.

Raw spectra were processed using in MaxQuant 1.6.6.0 (MQ) [34] and Perseus [35]. The data were searched against Human Uniprot Swissprot database, containing canonical and isoform proteins, version from 02.2019, and additionally the sequences of VSV-G, Nano-Luc and EPN were added.

MaxQuant search was performed with the default parameter set, including Trypsin/p protease specificity, max 2 missed cleavages, Met oxidation, Protein N-term acetylation and NQ deamidation as variable modifications and Carbamidomethyl Cys as a fixed modification, max 5 modifications per peptide, 1% PSM and protein FDR. The following options were turned on: second peptide, maxLFQ, match between runs. All runs were analyzed as independent experiments and processed in Perseus.

In Perseus, the protein group results were filtered for contaminants, reverse and “identified only by site” proteins. Only the proteins with maxLFQ values in at least 3 out of 7 LC-MS runs were used. For them, missing values were imputed from normal distribution with 0.3 intensity distribution sigma width and 1.8 intensity distribution center downshift. To quantify proteins in each sample, we used the iBAQ algorithm, implemented into MaxQuant software [36]. Normalization of each protein’s iBAQ value to the sum of all iBAQ values generates a relative iBAQ (riBAQ) value corresponding to the mole fraction of each protein [37]. GO analysis was performed in DAVID [38,39].

### 2.10. Molecular Modeling

Visualization of the EVs was created in CellPAINT 2.0 [40]. Individual sprites were created in Illustrate [41] based on 3D structures generated with trRosetta [42].

### 2.11. Estimation of Protein Numbers in EVs

The average EV particle mass was estimated, assuming the average EV diameter of 105 nm measured by TEM and EV density of 1.14 g/mL corresponding to 33% (*v*/*w*) sucrose fraction.

(1) $$m=\frac{4}{3}\pi r^{3}\cdot \rho =\frac{4\times \pi \times {\left(52.5\times 10^{-9}\;m\right)}^{3}}{3}\times 1140\;\frac{\mathrm{kg}}{m^{3}}=6.9\times 10^{-16\;}g$$

To determine the abundance of VSV-G, EPN and NanoLuc in EVs, we performed quantitative Western blot and luciferase assay experiments corrected by MS-MS analysis. To measure the amount of VSV-G protein contained in the EVs, we performed Western blot experiment with recombinant protein stands (Figure S4). To measure the amount of NanoLuc protein contained in the EVs, we performed luciferase assay with prokaryotic recombinant NanoLuc as standard (Figure S5). To measure the amount of EPN protein contained in the EVs, we used EPN/NanoLuc molar ratio estimated by MS/MS analysis (Table S2). As a result, the following concentrations were obtained: 65·10^−3^ µg VSV-G and 25·10^−3^ µg NanoLuc per µg of EVs in the case of co-transfection with pCMV-VSV-G, p-CMV-NanoLuc-Jun, and pCMV-EPN-Fos-C plasmids (EV_C); 150·10^−3^ µg VSV-G and 8·10^−3^ µg NanoLuc per µg of EVs in the case of co-transfection with pCMV-VSV-G, pCMV-NanoLuc-Vpr, and pCMV-EPN-p6 plasmids (EV_p6); 65·10^−3^ µg VSV-G and 60·10^−3^ µg NanoLuc per µg of EVs in the case of lentiviral vector pLX-NanoLuc-EPN-VSVG (EV_C(lenti)).

Therefore, we estimate the amount of the analyzed protein in a single EV according to the following formula:

$$N=\frac{protein\;amount\;in\;1\;mcg\;of\;vesicle\times vesicle\;mass\;(6.9\times 10^{-16\;}g)}{protein\;molecular\;weight}\times N_{A}\;\left(6.02\times 10^{23}\frac{1}{\mathrm{mol}}\right)$$

### 2.12. Quantification and Statistical Analysis

All experiments were carried out in triplicate (biological repeat). Each biological repeat for DLS and luciferase assay was additionally measured at least in triplicate (technical repeat). Statistical analyses of data were performed with GraphPad Prism 7. Data are represented as mean ± SD. For the comparison of differentially expressed proteins between cell-whole lysates and EVs, as well as across the EV subtypes, an ANOVA test was performed.

## 3. Results

### 3.1. Improving Loading Capacity of Protein Nanocages Preserves Their Capacity to Self-Assemble

Spike glycoprotein of the vesicular stomatitis virus (VSV-G) is commonly used for pseudotyping retroviral and lentiviral particles to facilitate membrane fusion with the target cells and enable their entry into mammalian cells. Recently, the usage of VSV-G has been started for producing the engineered vesicles and the subsequent delivery of the exogenous proteins into human cells [43,44]. Active protein loading inside the self-assembling EPN nanocages and their further encapsulation in VSV-G-containing EVs were realized via fusion of the 14 kDa Vpr to the delivered protein [28]. Naturally, Vpr interacts with the p6 domain of the HIV-GAG precursor, thereby packaging Vpr-fusion proteins into viral particles [45]. Upon an attempt to use Vpr for recombinant protein delivery into HIV-1 particles, Vpr-dependent G2 arrest activity and epigenetic disruption of heterochromatin were observed [46,47]. There are several reports of Vpr mutants with lower cytotoxicity [48], but harnessing this protein for therapeutic purposes is still under debate.

**Figure 1:**
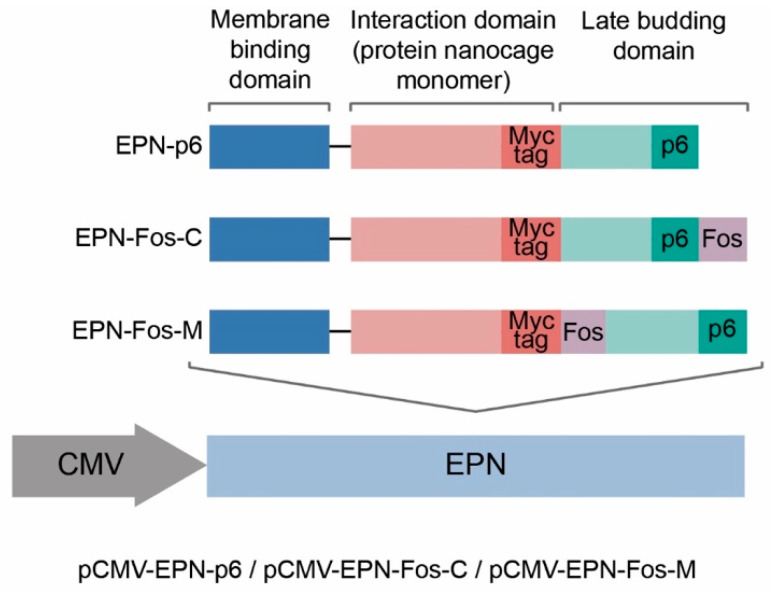
Construction of the pCMV-EPN vectors. Each EPN monomer consists of 3 functional domains: Membrane binding, Interaction and Late budding domain. EPN-Fos-C and EPN-Fos-M include additional Fos domain at the C-terminal of protein sequence and at the N-terminal of the Late budding domain, respectively. Fos domain provides active loading of EPN nanocages with the protein of interest fused with Jun domain. (The analogic constructs with T7 promoter were obtained in pET22-based prokaryotic vector).

Here, the previously reported EVs [28], containing nanocages with the p6 domain, are designated as “EV_p6”. To be able to use the EVs as a therapeutic agent, we replaced this toxic and oncogenic protein Vpr with the domain of the leucine zipper of the Jun protein, which forms a leucine zipper heterodimer with the domain of the Fos protein [49]. One of the substantial advantages of the Fos-Jun dimer compared with Vpr-p6 is its small size (4 kDa). Utilizing these domains instead of Vpr full-length protein allows us to fully exploit the loading potential of protein nanocages. We explored two new variants of EPN protein nanocage scaffolds with two different positions of the Fos domain—at the C-end of translated protein (EPN-Fos-C) or between the Interaction domain and the Late budding domain (EPN-Fos-M) (Figure 1).

**Figure 2:**
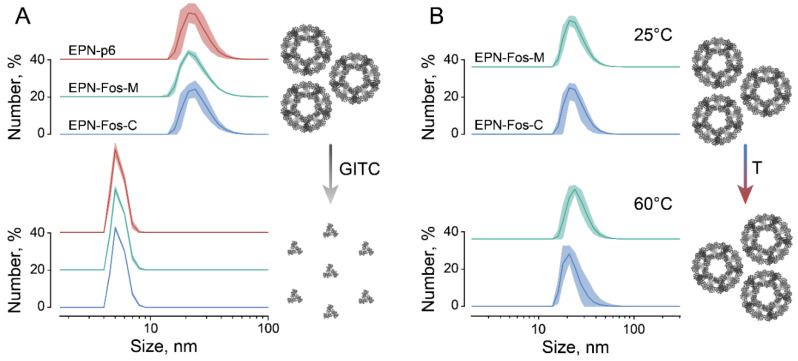
The stability of protein EPN nanocages containing Fos domain. (**A**) DLS experiments reveal measured hydrodynamic diameter of EPN-p6 (24.3 ± 1.0 nm), EPN-Fos-C (25.8 ± 0.7) and EPN-Fos-M (24.8 ± 0.2) (n = 3). After overnight incubation under denaturing conditions (3 M GITC) protein nanocages disintegrated into monomers. (**B**) Modified protein nanocages with Fos domain can withstand heating up to 60 °C.

EPN protein nanocages are highly ordered self-assembled protein dodecahedrons, consisting of 60 monomers, each provided with one p6 domain (“EPN-p6” Figure 1) [27]. To confirm the capability of the modified protein nanocages EPN-Fos-C and EPN-Fos-M to self-assemble into highly ordered structures, we performed the production of EPN in the prokaryotic system. Dynamic light scattering (DLS) measurements of modified protein nanocages with the Fos domain showed a monodisperse population of particles with a hydrodynamic diameter of 25 nm (Figure 2), which corresponds to the size of the original nanocages with only the p6 domain [27]. Additionally, to compare their stability with the original EPN-p6, the purified nanocages were incubated overnight in the presence of 3 M GITC (denaturing conditions) or in non-denaturing conditions. Compared to literature data, the dissociation of the original EPN-p6 and modified nanocages was observed only at 3 M GITC (Figure 2A). No disassembly was observed at 60 °C (Figure 2B) or in 2 M GITC (data not shown). Thus, we state that the Fos domain does not influence the ability of EPN nanocages to self-assemble. Assuming this with the fact that Fos and Jun domains are biocompatible, we believe that EPN-Fos can be considered to be a promising drug carrier in therapeutics development.

### 3.2. Production of Engineered EVs in Mammalian Cells

**Figure 3:**
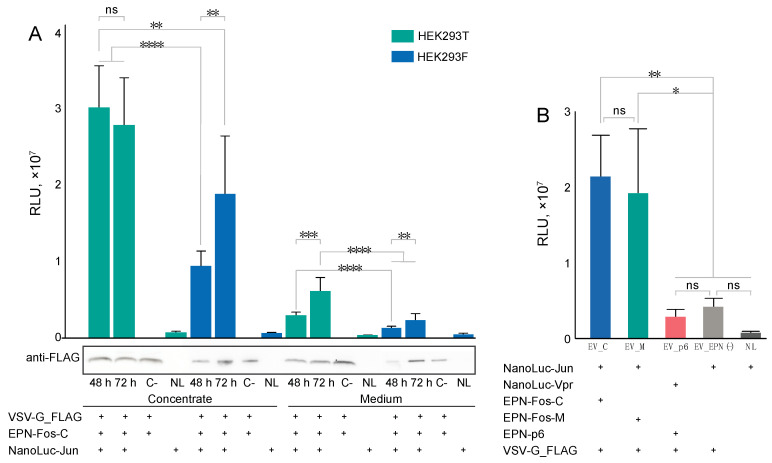
Expression level of EVs loaded with NanoLuc-Jun. (**A**) Optimization of EV production in eukaryotic cells. Data are shown for cells’ supernatant (Medium) before concentration steps and EVs concentrated with 100 kDa membrane (Concentrate). “48 h”, “72 h”—samples containing EVs with EPN nanocages loaded with NanoLuc-Jun collected after 48 h and 72 h of expression, respectively. “C-”—samples containing EVs with empty EPN nanocages (without NanoLuc-Jun), collected after 72 h of expression (negative control). (**B**) EV_C and EV_M samples produce the same level of EVs loaded with NanoLuc. Relative Light Units (RLU) were measured. EVs from cells-producer line were collected after 48 h or 72 h, concentrated and washed three times with PBS as described in Materials and Methods. “NL”—samples containing NanoLuc-Jun expressed without VSV-G and EPN for 72 h (control). Each experiment was performed in triplicate. Mean ± SD is shown. * *p* < 0.05, ** *p* < 0.01, *** *p* < 0.001, **** *p* < 0.0001, ns—not significant, two-tailed Student’s *t*-test. Utilization of each of the genetic constructs (pCMV-VSVG, pCMV-EPN-Fos-C, pCMV-EPN-Fos-M, pCMV-EPN-p6, pCMV-NanoLuc-Jun, pCMV-NanoLuc-Vpr) for the production of EVs is indicated as “+”.

The self-assembled nanocages loaded with protein cargoes could be delivered to target cells via engineered genetically encoded EVs. For a more reliable analysis of cargo encapsulation and delivery, we used NanoLuc as a model for potential protein pharmaceutics. First of all, we confirmed that fusion with the Jun domain will not interrupt the encapsulation of self-assembled nanocages loaded with NanoLuc-Jun in EVs. Using pCMV-VSV-G, pCMV-EPN-Fos-C and pCMV-NanoLuc-Jun for transient co-transfection of eukaryotic cells, we obtained the EVs loaded with NanoLuc and optimized EV production (Figure 3A). It was shown that both tested cell lines are able to produce EVs. Nevertheless, HEK293T cells are more suitable, as they have a higher level of EV production in a shorter time (48 h). Further, we showed that the use of EPN-Fos-M nanocages allows the production of the same level of EVs loaded with NanoLuc comparable to EPN-Fos-C (Figure 3B).

### 3.3. EV Production by Lentiviral Construction

**Figure 4:**
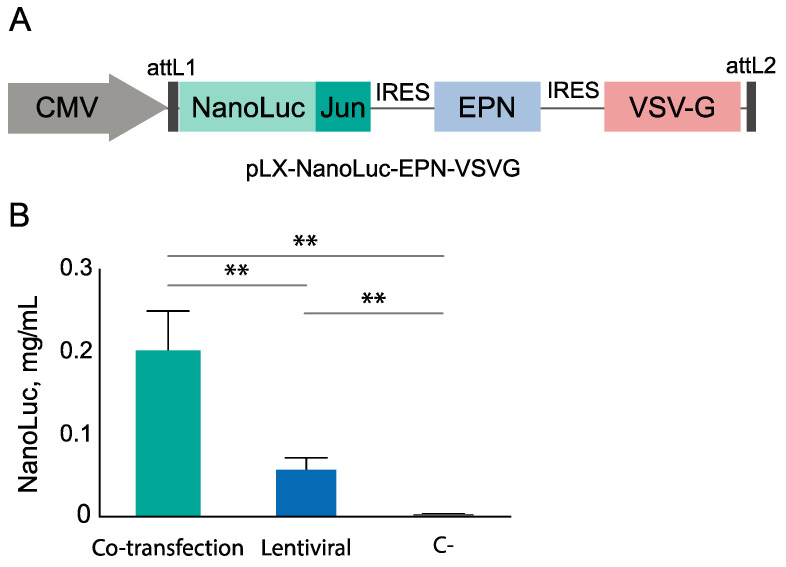
(**A**) Construction of the pLX-NanoLuc-EPN-VSV-G vector. NanoLuc-Jun/EPN/VSVG genes are separated via IRES sequences. (**B**) Comparison of EV production efficiency utilizing co-transfection of three vectors (pCMV-VSVG, pCMV-EPN-Fos-C, pCMV-NanoLuc-Jun) and utilizing lentiviruses. For each analysis, samples were concentrated with 100 kDa membrane and washed three times with PBS to remove NanoLuc which is not encapsulated in EVs. NanoLuc-Jun amounts in EVs were measured via NanoGlo assay, utilizing prokaryotic NanoLuc-Jun protein for calibration. ** *p* < 0.01, two-tailed Student’s *t*-test (n = 3).

In case of the genetically encoded EVs used as a therapeutic agent, it is advisable to use the single genetic construct. The co-transfection of eukaryotic cells with several genetic constructs leads to non-uniform distribution of vectors between cells. To ensure a broader standardization of EVs, we created the multicistronic construct based on a lentiviral vector pLX (Figure 4A). Other advantages of a lentiviral vector are the possibility to deliver gene information into dividing and non-dividing cells and to create a stable cell producer line for EVs, which is significantly cost-effective. These features provide the ability to mediate long-term therapeutic transgene expression not only *in vitro* but also in a patient’s body. We analyzed the total yield of NanoLuc encapsulated into EVs, both those produced in co-transfected cells and with a lentiviral construct utilized. Although the overall yield of the encapsulated NanoLuc is falling in the case of lentiviruses (Figure 4), lentiviral transduction can be applied for further EV production as it allows for the theoretically maximum cargo filling of each individual EV.

### 3.4. Isolation and Characterization of Genetically Encoded EVs

To further characterize the engineered EVs and compare them with the previously published data, we obtained the EVs with modified EPN-Fos-C, EPN-Fos-M nanocages and EVs produced with a lentiviral construction (EPN_C(lenti)) (Table S1). As a control for the non-specific encapsulation of the targeted protein, we analyzed co-transfection without EPN nanocages (EV_EPN(-)). As a control for the natural EVs constitutively produced by cells, where NanoLuc can be encapsulated non-specifically, we used transfection with pCMV-NanoLuc-Jun only (designated NLuc). Vesicles obtained in eukaryotic producer cells are often contaminated with various proteins and self-vesicles from the media serum. Further analysis of the genetically encoded EVs requires ultra-purified preparations, i.e., density separation of EVs being “a gold standard” for vesicle isolation [50]. Prior to gradient centrifugation, we performed several steps of differential centrifugation, concentration of the final supernatant and its washing at 100 kDa membrane to remove media proteins including soluble NanoLuc, which is not encapsulated into EVs. The presence of the genetically encoded EVs was checked with a luciferase assay. For all types of vesicles, except “NLuc”, luciferase was enriched in fractions with 25–40% (*w*/*v*) sucrose (Figure S2). We measured the size of the highly purified EVs utilizing the dynamic light scattering method (Figure S3 and Table 1). The average diameter of EVs loaded with EPN was ~170 nm and did not differ significantly (*p* > 0.5), while unloaded EV_EPN(-) were smaller (*p* < 0.01) and had an average diameter of 150 nm.

**Figure 5:**
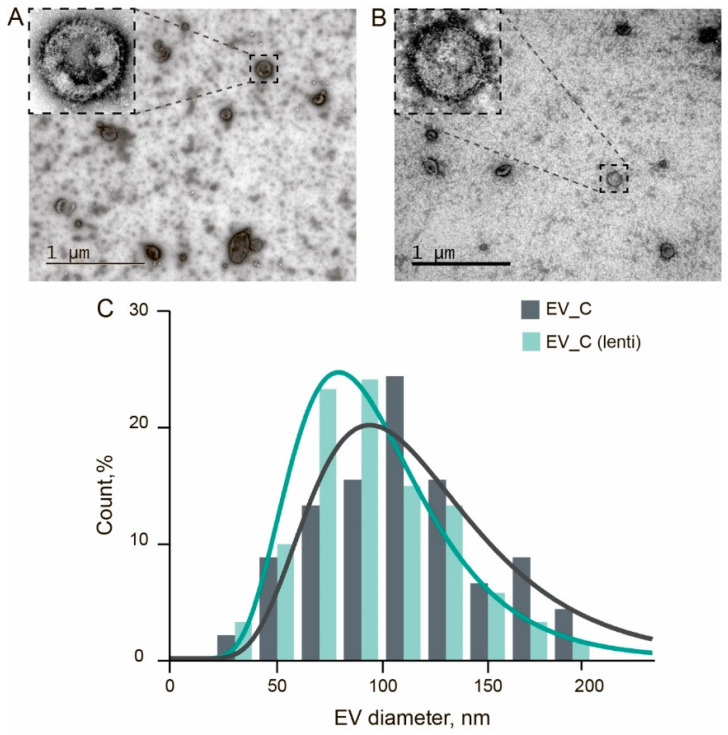
TEM analysis of the EVs. (**A**,**B**)—TEM images of EVs obtained with co-transfection and lentiviral constructs, respectively. (**C**) Size distributions of the EVs according to TEM images.

The TEM images of the samples EV_C and EV_C(lenti) are shown in Figure 5A,B. Most particles demonstrated the cup-shaped morphology, which is typical of the EVs imaged by negative staining TEM. Up to one-third of the particles had visible VSV-G spikes protruding from the vesicle membranes, as shown in the insets. The mean thickness of this “shell” was 12 ± 2 nm (mean ± SD). The mean sizes of the EV_C were 110 ± 40 nm and of EV_C(lenti) were 100 ± 40 nm. The histograms of the size distributions are shown in Figure 5C.

**Figure 6:**
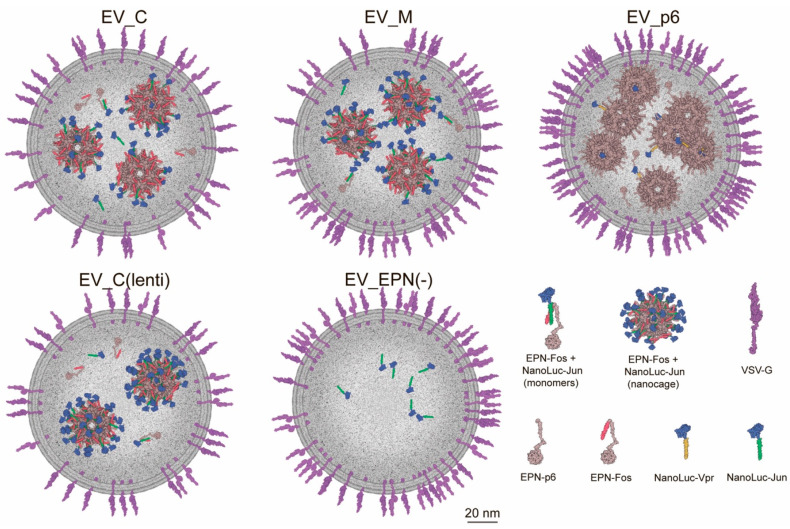
Two-dimensional modeling of EV_C, EV_M, EV_p6, EV_C(lenti) and EV_EPN(-): 1/10 of all proteins of interest shown in each case.

To calculate the amounts of VSV-G and NanoLuc-Jun in each EV, we first performed a quantitative measurement of VSV-G by immunoblotting and NanoLuc measurement by luciferase assay using the purified recombinant proteins as standards. According to this model, we estimated the quantity of key recombinant proteins and nanocages in EVs (Table 2). The amount of NanoLuc molecules in every single EPN nanocage was significantly higher in EV_C as compared with EV_p6 (15 and 1.5 molecules, respectively), which may be due to the small molecular size of the Jun domain and its flexibility. However, EV_C is inferior in terms of loading capacity to EV_C(lenti). In EV_C(lenti), almost all sites on the surface of EPN nanocages were occupied with NanoLuc cargo (40 out of 60). This observation confirms the importance of the uniform distribution of genetic constructs among the cells for producing the vesicles loaded with cargo proteins. To illustrate different EVs, filled with cargo protein (NanoLuc) and nanocages, we obtained a 2D model (Figure 6).

### 3.5. Delivery of Protein Cargo to Target Cells

**Figure 7:**
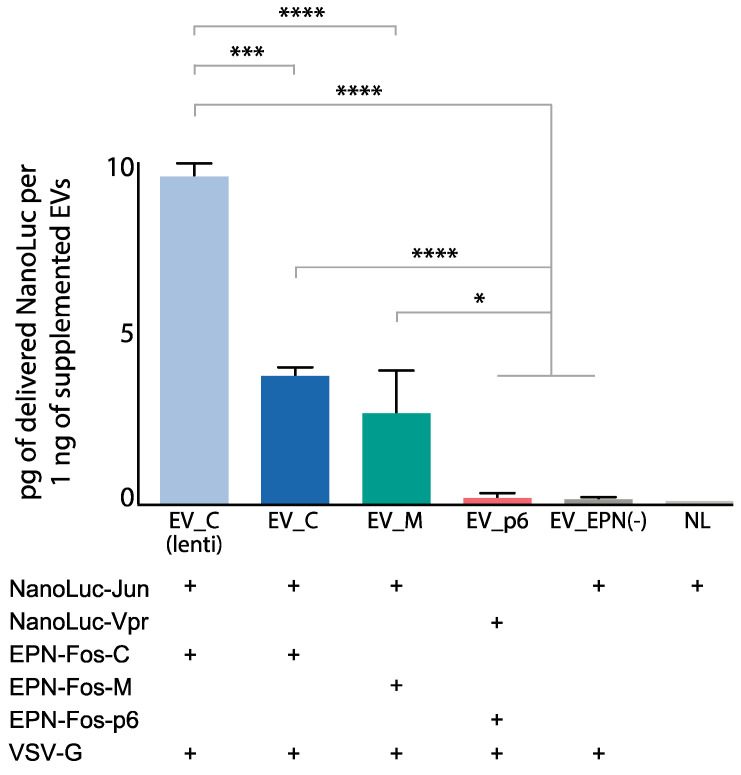
The level of delivered NanoLuc in cells after 2 h of incubation. Highly purified EVs containing 50 pg of NanoLuc were applied to 40,000 target cells. The amount of added EVs was normalized to NanoLuc portion in each EV sample. Data are mean ± SD, 3 biological repeats were used. * *p* < 0.05, *** *p* < 0.001, **** *p* < 0.0001, two-tailed Student’s *t*-test.

To confirm the functionality of EVs with the novel Fos domain and lentiviral EVs, we analyzed their ability to deliver cargo proteins into target cells. Each sample was diluted according to NanoLuc portion before the incubation with target cells. Delivery efficiency was quantified via luciferase assay. We found that EV_C and EV_M vesicles delivered NanoLuc-Jun more effectively than EV_p6 (Figure 7), indicating the improvement of the initial EV capacity. It should be mentioned that the lentiviral construct showed the same efficiency as the EVs obtained via a co-transfection when adding an equal amount of NanoLuc, loaded into EVs (Figure S6). However, EVs_C(lenti) contained more than twice as much NanoLuc in the equal amount of EV preparation. Thus, considering the total amount of the EVs supplemented to the target cells, it is evident that EV_C(lenti) allow the delivery of more NanoLuc. Thereby, using the lentiviral construct is the most effective method of loading a protein cargo into engineered EVs and delivering it to the target cell.

### 3.6. Proteome Profiling of Engineered EVs

To better understand the protein composition of the obtained vesicles, proteome analysis of highly purified vesicles was performed. An equal amount of input proteins for three independent preparations for each type of vesicle was trypsinized and analyzed by LC-MS/MS. To evaluate protein abundance, we further used intensity-based absolute quantification (iBAQ) [51]. For this analysis, the sum of peptide intensities referring to one protein is normalized to the number of the theoretically possible peptides. The relative iBAQ then represents the relative proportion of each protein in the sample (Table S2). We first compared the relative iBAQ values of recombinant proteins VSV-G, EPN and NanoLuc. The amount of VSV-G was slightly elevated in EV_p6 and EV_EPN(-) vesicles as compared to the improved vesicles containing the Fos domain (~20% versus ~10%). At the same time, the EPN protein, as expected, was not detected in EV_EPN(-) and was significantly decreased in these improved vesicles assembled with the Fos domain (4–10% versus 15–25%). Apparently, the presence of an additional domain partially obstructs EPN expression, nanocage assembly or its packaging into vesicles. However, the amount of the delivered NanoLuc was significantly elevated in the newly designed vesicles (~2–4%), as compared to EV_p6 and EV_EPN(-) vesicles (<0.5%). These data are in good agreement with Western blot and luciferase assay analysis, revealing an increased NanoLuc loading and a decreased amount of VSV-G within vesicles obtained with the newly developed system utilizing Fos–Jun interaction instead of Vpr-p6.

To gain further functional insight into the proteomic cargo in purified EVs, we performed gene ontology (GO) analysis using the DAVID database v6.8. Firstly, we obtained the whole-cell proteomes of all cells, producing the investigated vesicles. Secondly, using whole-cell proteomes as a background for vesicle proteomes, we obtained the statistically significant enrichment only for 5-12 proteins for EV_C, EV_M, EV_C(lenti), EV_EPN(-) and 37 proteins for initial EV_p6 (Table S3). The identified proteins were strongly enriched in extracellular exosome, extracellular matrix, and extracellular space cellular components, further confirming the efficient EV isolation. It should be noted that since the initial EV_p6 had a greater amount of significantly elevated proteins compared to the whole-cell lysate, more GO categories were identified for them. For EV_p6, mainly cellular components related to extracellular exosome, matrix, space, and vesicle were identified, but the adhesion, cytoskeleton, and membrane categories were also significantly enriched.

**Figure 8:**
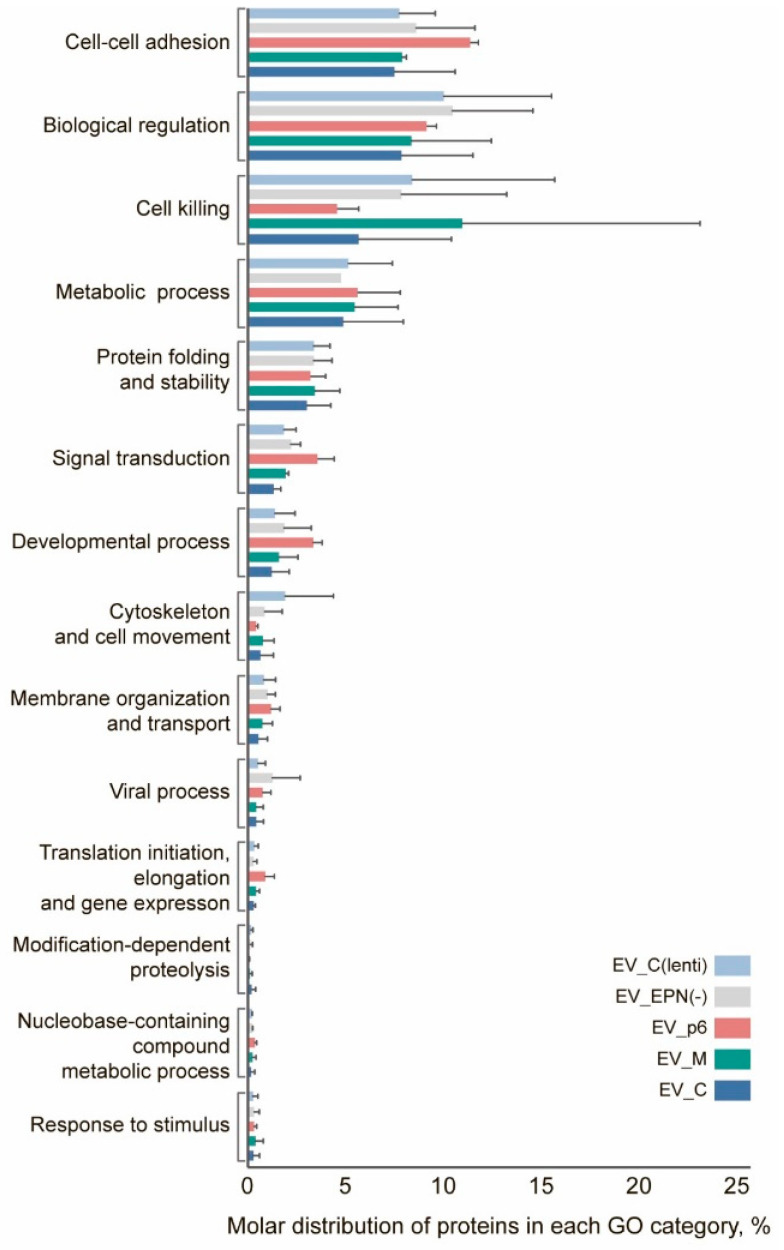
Gene ontology enrichment analysis of the EV proteins identified using the DAVID database. No significant differences among EV samples were found as determined by two-way ANOVA.

Next, we selected 135 high confidence proteins that were quantified in all three independent proteomic assays performed on different biological batches for at least one type of vesicle (Table S2). Interestingly, among the enriched GO terms pointed out by this analysis, several biological processes were highlighted. They were related not only to membrane organization, transport, cell-cell adhesion, cytoskeleton and cell movement, but also cell killing, biological regulation, response to stimulus, modification-dependent proteolysis, signal transduction, viral process, developmental process, metabolic process, gene expression and protein folding. Based on the relative number of protein molecules calculated by iBAQ analysis, there was no statistically significant difference between all types of the investigated vesicles, neither in individual protein abundance nor in the distribution of proteins classified into GO categories (Figure 8). At the same time, surprisingly, the majority of abundant proteins were enriched in biological processes, related not only to cell–cell adhesion, but also to biological regulation, cell killing, metabolic process, and protein folding.

## 4. Discussion

In this study, we put forward an improved method of protein pharmaceutics delivery enveloped in the engineered EVs. The proposed method aims to provide an easy to modulate, effective and low-cost machinery to envelope protein cargoes into EVs and to deliver them precisely to target cells. We demonstrate that active loading of the engineered EVs applying modified EPN-Fos nanocages increases the efficacy of loading cargo inside EVs and delivering it to target cells.

The development of novel protein delivery systems is at the focus of many research groups. Two major tasks yet to be resolved are to prevent protein degradation and to achieve its specific delivery into the target cells. However, despite a wide palette of protein drug carriers existing today, many of them are not approved for human therapy due to their possible toxicity, while others are used very rarely due to their high cost. Utilizing viral proteins for pharmaceutic envelopes seems to be a highly effective strategy, but it may raise some concerns, largely due to VSV-G immunogenicity. Research investigating the potential to reduce this immunogenicity via pseudotyping with different glycoprotein subtypes is currently underway [52,53,54], but it still remains a challenge [55]. Alternatively, new strategies for the efficient production of designer exosomes, for example, the utilization of recombinant CD63 instead of VSV-G, are proposed [56]. Thus, the future nanocage-containing EVs could be obtained with a non-toxic and non-immunogenic analog of VSV-G. Another biosafety question is concerned with the Vpr protein used for the active load of cargo inside the previously described nanocage-containing EVs.

Here, we replaced this toxic protein with the leucine zipper domain of the Jun protein, which is present in normal human cells. Although the whole Jun protein is marked as a proto-oncogene [57], we took only a small, 4 kDa part of this protein—its leucine zipper. The investigated engineered EVs deliver their cargo into the cytoplasm of target cells due to VSV-G interaction with low density lipoprotein receptors on the cell surface and subsequent envelope fusion with early endosome membrane [58]. Noncovalent Fos-Jun binding allows for a dissociation of a nanocage and cargo protein in a recipient cell without nanocage disassembling, providing the targeted delivery of protein pharmaceuticals. This property turns out to be useful, for example, for the delivery of transcription factors. Comparison with the recently published results, reporting the development of Gectosome—split GFP system [44], suggests that the engineered nanocage-containing EVs allow more pharmacological protein molecules loaded into the same vesicle. The EVs developed here demonstrate that the ratio of VSV-G to delivered protein reaches 1:2 in the case of utilizing the lentiviral construct, while the ratio of VSV-G to delivered protein is 6:1 in the case of Gectosomes [44]. The total amount of NanoLuc molecules in every single EPN nanocage was significantly higher in EV_C as compared with the initial EV_p6.

One possible reason for such an improvement might lie in the Fos domain structure. This domain, together with its glycine-linker, forms a “flexible hand”, which allows it to direct the binding site of loading protein to the outer surface of EPN and therefore facilitate a more efficient interaction with the Jun domain and cargo protein than the p6 region. Fos-linker flexibility and length allow it not to load all the delivered protein inside the nanocage, but to place it outside. Therefore, in the case of Fos-Jun nanocage-containing EVs, we are not limited with the volume of the EPN nanocage, which is significantly less than the volume of the total vesicle. Still, the possibility to deliver larger proteins utilizing the modified nanocage-containing EVs requires further studies. Meanwhile, the investigated EVs containing EPN nanocages seem to be a promising platform for the targeted delivery of small protein therapeutic agents, such as peptides, required to enhance antigen presentation. If the highly specific delivery of engineered EVs is required, there are two major strategies to achieve it: (i) antibodies, anchored into the EV membrane [59]; (ii) VSV-G replacement with other viral glycoproteins, such as RVG, which will redirect EV tropism [60]; (iii) using the targeting peptides, which are mostly fused with transmembrane proteins [26], and, in the case of 5–9 a.a. peptides, they could be fused with VSV-G molecules without any loss of activity [61]. It should be mentioned that in spite of more efficient cargo loading and protein delivery, the use of lentiviral genetic constructs with two IRES sequences resulted in a significant decrease in EV yield compared to the co-transfection system. In the future, it may be necessary to optimize EV production by constructing lentiviral vectors with several independent internal promoters. The EPN sequence of the late budding domain comprising the p6 domain could also be modified and partially reduced. Originally, this domain recruited ESCRT machinery to catalyze the final membrane fission step required for release from the cell. The utilization of VSV-G can reduce dependence on ESCRT machinery recruiting.

In general, EVs are described as a very heterogeneous population with hundreds of associated markers [62]. We obtained mass spectrometry data for the investigated nanocage-containing vesicles and gene ontology analysis revealed that the majority of abundant proteins were enriched in a variety of biological processes, such as membrane organization, transport, cell–cell adhesion, cytoskeleton, cell movement, cell killing, biological regulation, response to stimulus and signal transduction. The presence of some proteins like cell–cell adhesion, cytoskeleton and membrane organization are essential for vesicle formation, while other non-structure proteins, like elements with protein folding activity, can be very useful for further delivery applications, since they could be helpful in maintaining the right folding of therapeutic proteins inside the engineered EVs.

Additionally, we show that a uniform distribution of genetic material in the case of a lentiviral construct instead of co-transfection with several plasmids leads to a more effective loading of therapeutics into the engineered EVs. This is an auspicious observation for future clinical applications of EVs. Utilizing the autologous long-living cells for transduction with lentiviral constructs makes it possible to obtain a cell population capable of producing engineered EVs *in vivo*. Future advances could spur the shift towards personalized medicine and give a chance to more effective therapy of autoimmune diseases and cancer.

## Figures and Tables

**Table 1:** The hydrodynamic diameter of EVs. Data were obtained in three biological replicates and indicate the mean diameter ± SD of size distribution estimated by dynamic light scattering, analyzed by t-test. The average diameter of EV_C, EV_M and EV_p6 was different from EV_EPN(-) (*p* < 0.01). Sizes of EV_C, EV_M, EV_p6 and EV_C(lenti) were not significantly different.

Sample	EV_C	EV_M	EV_p6	EV_EPN(-)	EV_C(lenti)
Size determined by DLS, nm	174 ± 45	176 ± 59	171 ± 57	150 ± 38	168 ± 45

**Table 2:** The estimated number of recombinant protein molecules per single vesicle. The numbers of VSV-G proteins are derived from the quantitative Western blotting results (Figure S4), the numbers of NanoLuc proteins are derived from the luciferase assay measurement (Figure S5) and the numbers of EPN proteins are derived from the EPN/NanoLuc molar ratio estimated by MS/MS analysis (Table S2). Data represent the mean of three biological replicates ± SD.

EV Samples	EV_C	EV_M	EV_p6	EV_(EPN-)	EV_C(lenti)
VSV-G, molecules	450 ± 50	700 ± 80	1000 ± 90	850 ± 80	450 ± 40
NanoLuc, molecules	430 ± 50	520 ± 50	110 ± 30	70 ± 30	1000 ± 80
EPN, molecules	1700 ± 600	1500 ± 500	4300 ± 1000	-	1500 ± 400
Assembled nanocages	28 ± 10	25 ± 8	71 ± 16	-	25 ± 7
NanoLuc, molecules in a single nanocage	15 ± 6	21 ± 7	1.5 ± 0.5	-	40 ± 12

## Data Availability

The mass spectrometry proteomics data presented in this study are available as Supplementary Tables S2 and S3. All other data are available on request from the corresponding author.

## Supplementary Materials

The following are available online at https://www.mdpi.com/article/10.3390/pharmaceutics13060768/s1, Figure S1. Schematic illustration showing the production, assembly, and release of EVs. Produced EVs, containing EPN nanocages, loaded with NanoLuc, were delivered to target cells. Figure S2. Luciferase activity in EVs fractionated in sucrose density gradient. Sucrose gradient fractions were analyzed for the presence of NanoLuc luciferase via NanoGlo assay. Fractions containing 25 to 40 % sucrose were identified as enriched in EVs. NanoLuc, which was not loaded into EVs, was found in the pellet. Figure S3. Characterization of highly purified EVs. The size distributions of EVs measured by dynamic light scattering (DLS). Each measurement was carried out in triplicate. Figure S4. Quantitative Western blotting analysis of the amounts of VSV-G-FLAG in highly purified EVs with equilibrated portion of VSV-G-FLAG. A known amount of recombinant proteins was used as standards for quantitation. Gel image is representative of 3 individual experiments. ”NLuc” sample corresponds to the same fraction of sucrose gradient in which all EVs were found (25–40% sucrose) and was loaded at the maximum volume. Lines with EVs were loaded with following amount of total protein: 30 ng of EV_C, 20 ng of EV_M, 13 ng of EV_p6, 17 ng of EV_EPN(-), 30 ng of EV_C(lenti). Figure S5. Estimated amounts of NanoLuc cargo protein (ng) in 1 μg of EVs. EVs were purified through sucrose gradient centrifugation. NanoLuc amounts in EVs were measured via NanoGlo assay, utilizing prokaryotic NanoLuc-Jun or NanoLuc-Vpr proteins for plotting a calibration curve. All measurements were taken in triplicate. Figure S6. The level of delivered NanoLuc in cells after 2 h of incubation. Highly purified EVs with loaded NanoLuc were applied to 40000 target cells. The amount of added EVs was normalized to NanoLuc portion in each EVs sample. Data are mean ± SD, 3 biological repeats were used. Table S1. List of plasmids used for EVs production in HEK293T cell line. Table S2. Qualitative proteomic analysis of the purified vesicles EV_C, EV_M, EV_p6, EV_EPN(-) and EV_C(lenti). Table S3. Proteomic analysis of proteins significantly enriched in the EV lysates compared to the whole cell lysates. Gene Ontology cellular component term enrichment analysis.
