# Supplementary material for: Reprogramming Extracellular Vesicles for Protein Therapeutics Delivery
Source: Pharmaceutics. 2021 May 21;13(6):768. doi: 10.3390/pharmaceutics13060768 (PMC8224366; doi:10.3390/pharmaceutics13060768)
Supplement: Supplementary file 1 [file pharmaceutics-13-00768-s001.zip › pharmaceutics-1207430 sup for xml.pdf]

pharmaceutics

MDPI

Supplementary Materials: Reprogramming Extracellular Vesicles for Protein Therapeutics Delivery

Leyla A. Ovchinnikova, Stanislav S. Terekhov, Rustam H. Ziganshin, Dmitriy V. Bagrov, Ioanna N. Filimonova, Arthur O. Zalevsky and Yakov A. Lomakin

Table S1. List of plasmids used for EV production in HEK293T cell line.

№	Name	Plasmid	DNA Amounts, µg per T75 Flask
1.	EV_C	pCMV-NanoLuc-Jun	7.5
		pCMV-EPN-Fos-C	13.5
		pCMV-VSVG	1.5
2.	EV_M	pCMV-NanoLuc-Jun	7.5
		pCMV-EPN-Fos-M	13.5
		pCMV-VSVG	1.5
3.	EV_p6	pCMV-NanoLuc-Vpr	7.5
		pCMV-Epn-p6	13.5
		pCMV-VSVG	1.5
4.	EV_EPN(-)	pCMV-NanoLuc-Jun	7.5
		pCMV-VSVG	1.5
5.	NLuc	pCMV-NanoLuc-Jun	1.5
6.	EV_C(lenti)	pLX-NanoLuc-Jun-EPN-C-VSVG	22.5

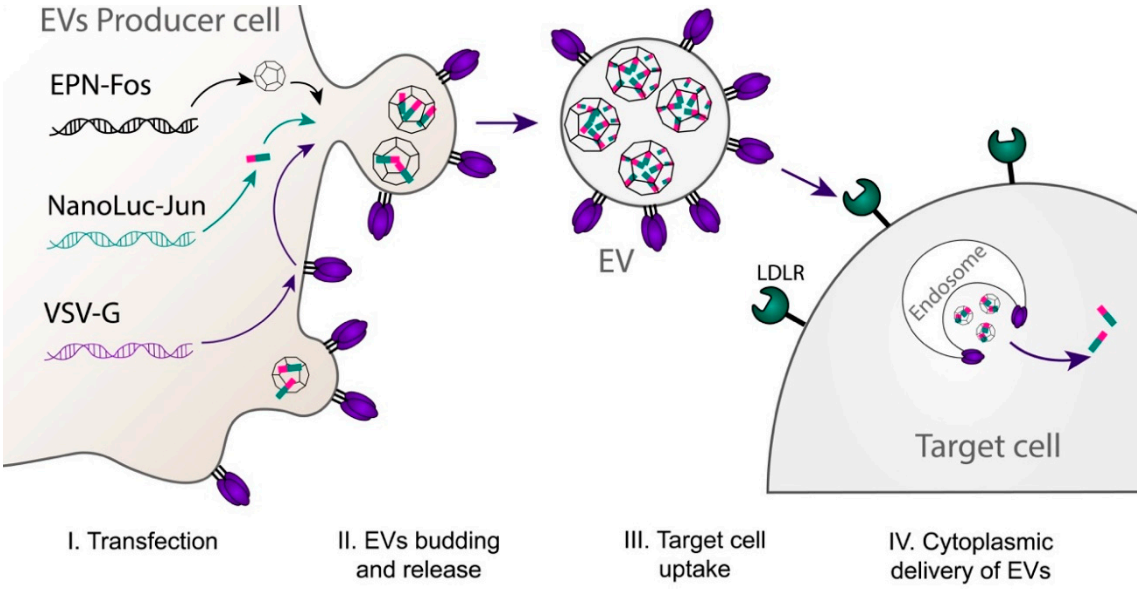

Figure S1. Schematic illustration showing the production, assembly, and release of EPNs incorporating NanoLuc and VSV-G proteins. Produced EVs, containing EPN nanocages, loaded with NanoLuc, were delivered to target cells.

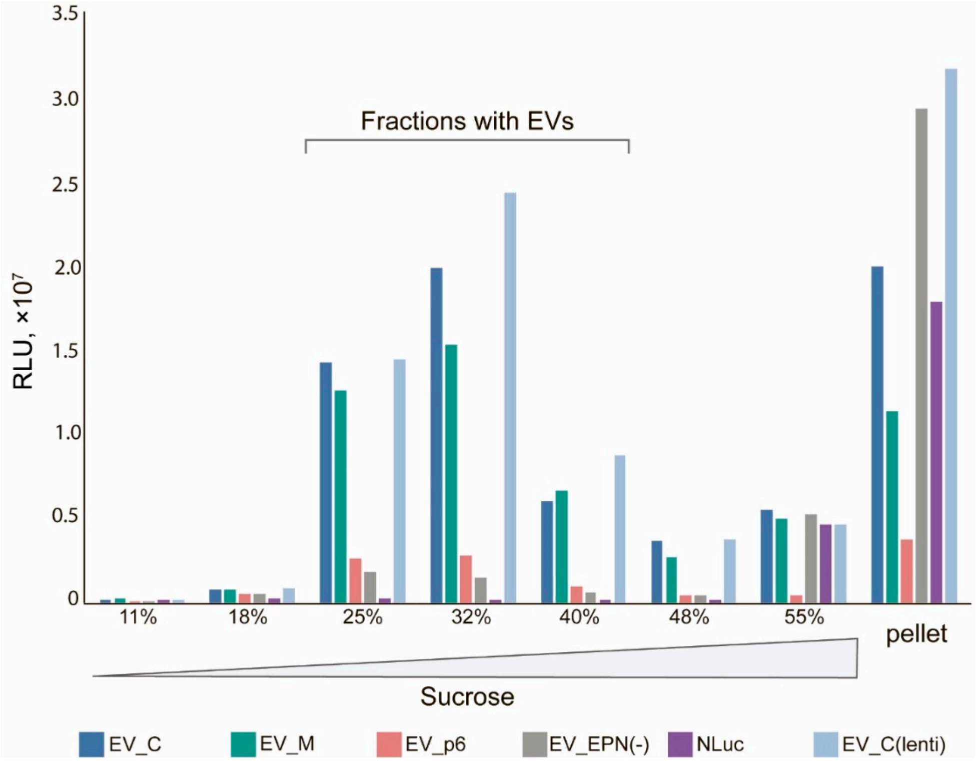

Figure S2. Sucrose gradient fractions were analyzed for the presence of NanoLuc-Jun luciferase via NanoGlo assay. Fractions containing 25 to 40% sucrose were identified as enriched in EVs. NanoLuc-Jun, which was not loaded into EVs, was also found in the pellet.

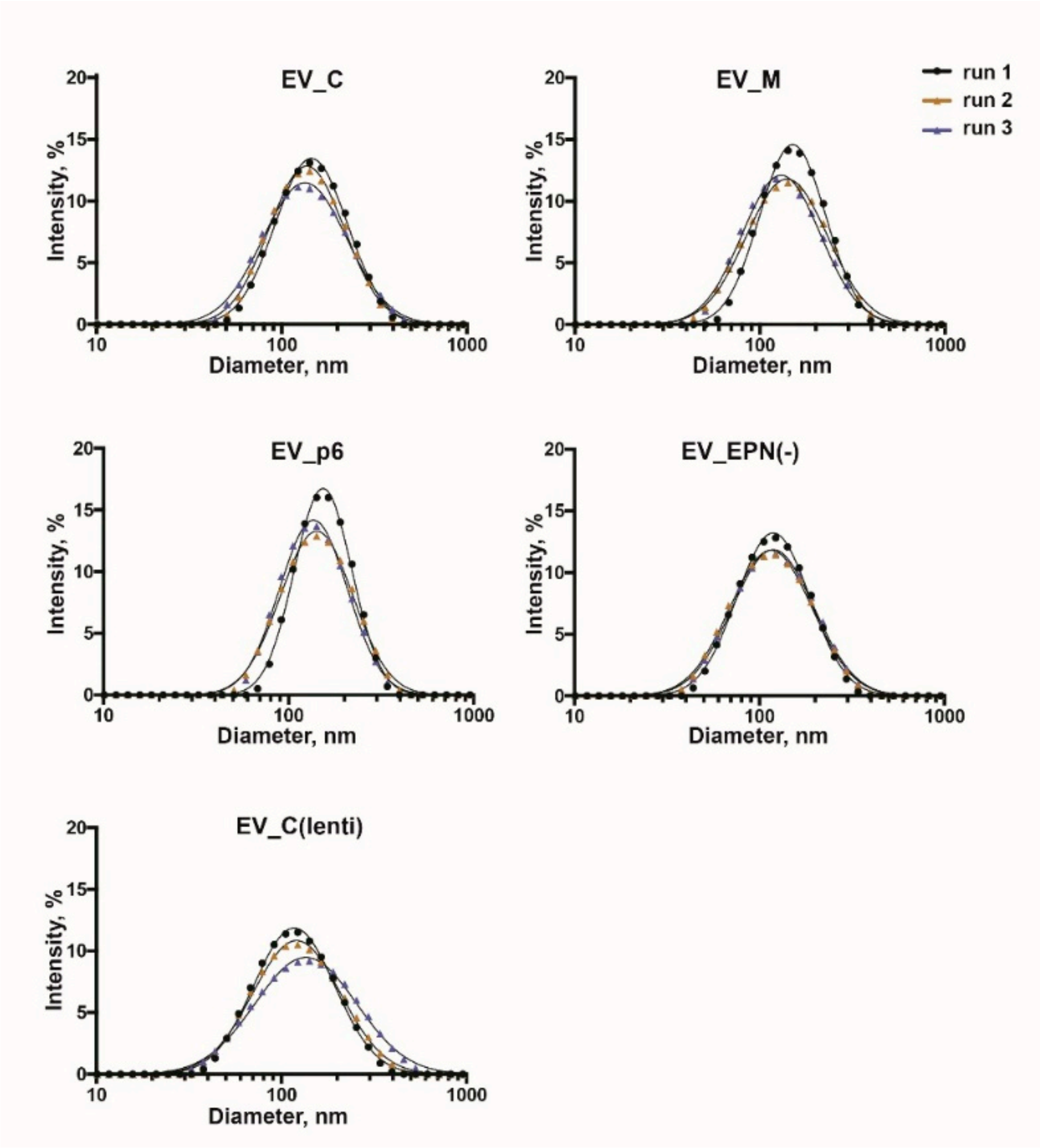

Figure S3. Characterization of highly purified EVs. The size distributions of EVs measured by dynamic light scattering (DLS). Each measurement was carried out in triplicate.

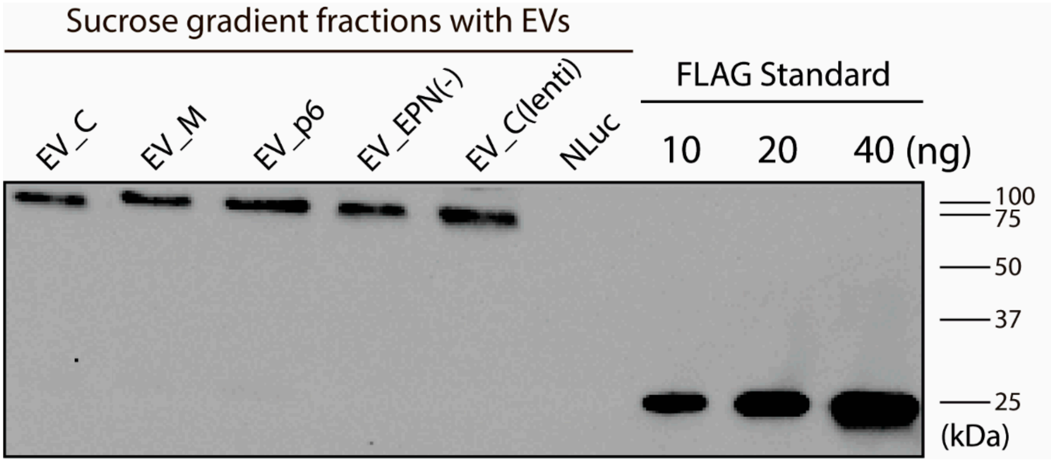

Figure S4. Quantitative Western blotting analysis of the amounts of VSV-G-flag in highly purified EVs with equilibrated portion of VSV-G-FLAG. A known amount of recombinant proteins was used as a standard for quantitation. Gel image is representative of 3 individual experiments. ″NLuc″ sample corresponds to the same fraction of sucrose gradient in which all EVs were found (25–40% sucrose) and was loaded at the maximum volume. Lines with EVs were loaded with

following amounts of total protein: 30 ng of EV_C, 20 ng of EV_M, 13 ng of EV_p6, 17 ng of EV(EPN-), 30 ng of EV_C(lenti).

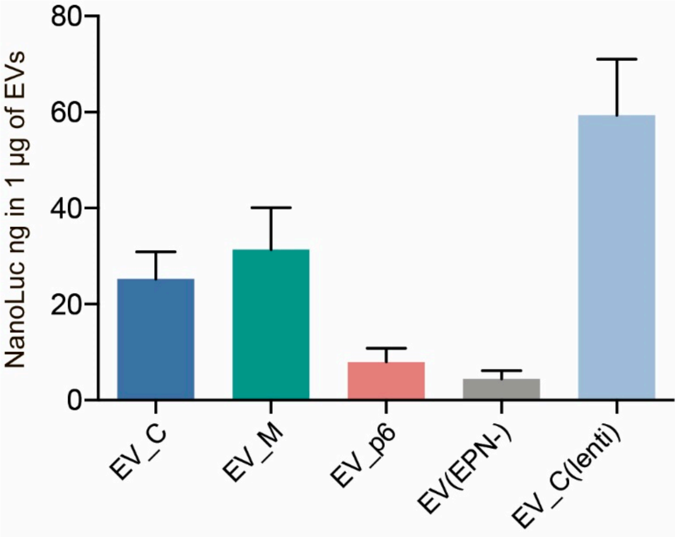

Figure S5. Estimated amounts of NanoLuc-Jun cargo protein (ng) in 1 µg of EVs. EVs were purified through sucrose gradient centrifugation. NanoLuc-Jun amounts in EVs were measured via NanoGlo assay, utilizing prokaryotic NanoLuc-Jun protein for plotting a calibration curve. All measurements were taken in triplicate.

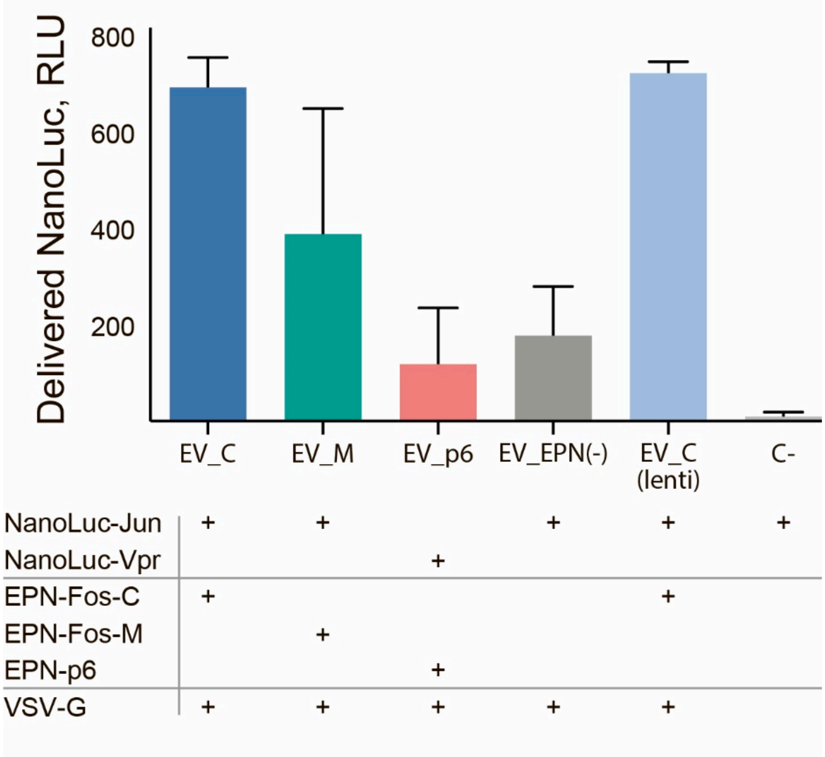

Figure S6. The level of delivered NanoLuc in cells after 2 h of incubation. Highly purified EVs with loaded NanoLuc were applied to 40,000 target cells. The amount of added EVs was normalized to NanoLuc portion in each EV sample. Data are mean ± SD, 3 biological repeats were used.
